# From nighttime light exposure to menstrual health: a critical review of evidence, mechanisms, and nursing interventions

**DOI:** 10.3389/frph.2026.1738574

**Published:** 2026-02-26

**Authors:** Junfen Hu, Suna Li, Xiaohui Yu, Lei Dai

**Affiliations:** 1 Department of Hepato-Pancreato-Biliary Surgery, Ningbo Medical Center Lihuili Hospital, The Affiliated Lihuili Hospital of Ningbo University, Ningbo, Zhejiang, China; 2 Department of Day Ward, Ningbo Medical Center Lihuili Hospital, The Affiliated Lihuili Hospital of Ningbo University, Ningbo, Zhejiang, China; 3 Department of Emergency, Ningbo Medical Center Lihuili Hospital, The Affiliated Lihuili Hospital of Ningbo University, Ningbo, Zhejiang, China

**Keywords:** circadian rhythm, epidemiology, female menstrual cycle, nighttime artificial lighting, nursing intervention

## Abstract

With the rapid progression of urbanization and the widespread adoption of nocturnal work and lifestyle patterns, artificial light at night (ALAN) has emerged as a significant environmental factor impacting women's health. Current research suggests that exposure to artificial light disrupts human circadian rhythms, potentially leading to irregular menstrual cycles, extended cycle durations, and altered hormone levels in women, thereby elevating reproductive health risks. This paper reviews epidemiological evidence concerning the impact of ALAN on menstrual cycles, critically evaluating the strength and limitations of evidence derived from different study designs. It further analyzes the mechanisms through which key parameters—including light intensity, spectral composition, and duration of exposure— influence circadian rhythms and endocrine systems, while explicitly noting that these mechanisms are largely derived from animal models and must be extrapolated to humans with caution. Furthermore, by integrating recent findings from nursing research, we propose evidence-based, tiered intervention strategies, encompassing health education, personalized lighting management, and lifestyle modifications, to mitigate potential disruptions caused by ALAN. The review aims to provide theoretical support and practical guidance for clinical nursing practices and public health policy formulation, and to highlight priority directions for future research.

## Introduction

With the rapid advancement of contemporary society, artificial light at night (ALAN) has emerged as a pervasive environmental phenomenon on a global scale. Whether in urban streets, office buildings, or residential homes, the ubiquity of artificial lighting has substantially extended human activity hours, facilitating the development of a 24-hour society (1). The extensive adoption of LED lighting and electronic screens—including smartphones, tablets, and computers—has markedly increased nighttime exposure to high-intensity, short-wavelength blue light (2, 3). However, this convenience is accompanied by latent health risks. Empirical evidence indicates that ALAN not only disrupts circadian rhythms, which have evolved over the course of human evolution, but also elevates the risk of various chronic diseases, including metabolic disorders, cardiovascular diseases, cancer, and mental health issues (1, 4, 5).

**Figure 1:**
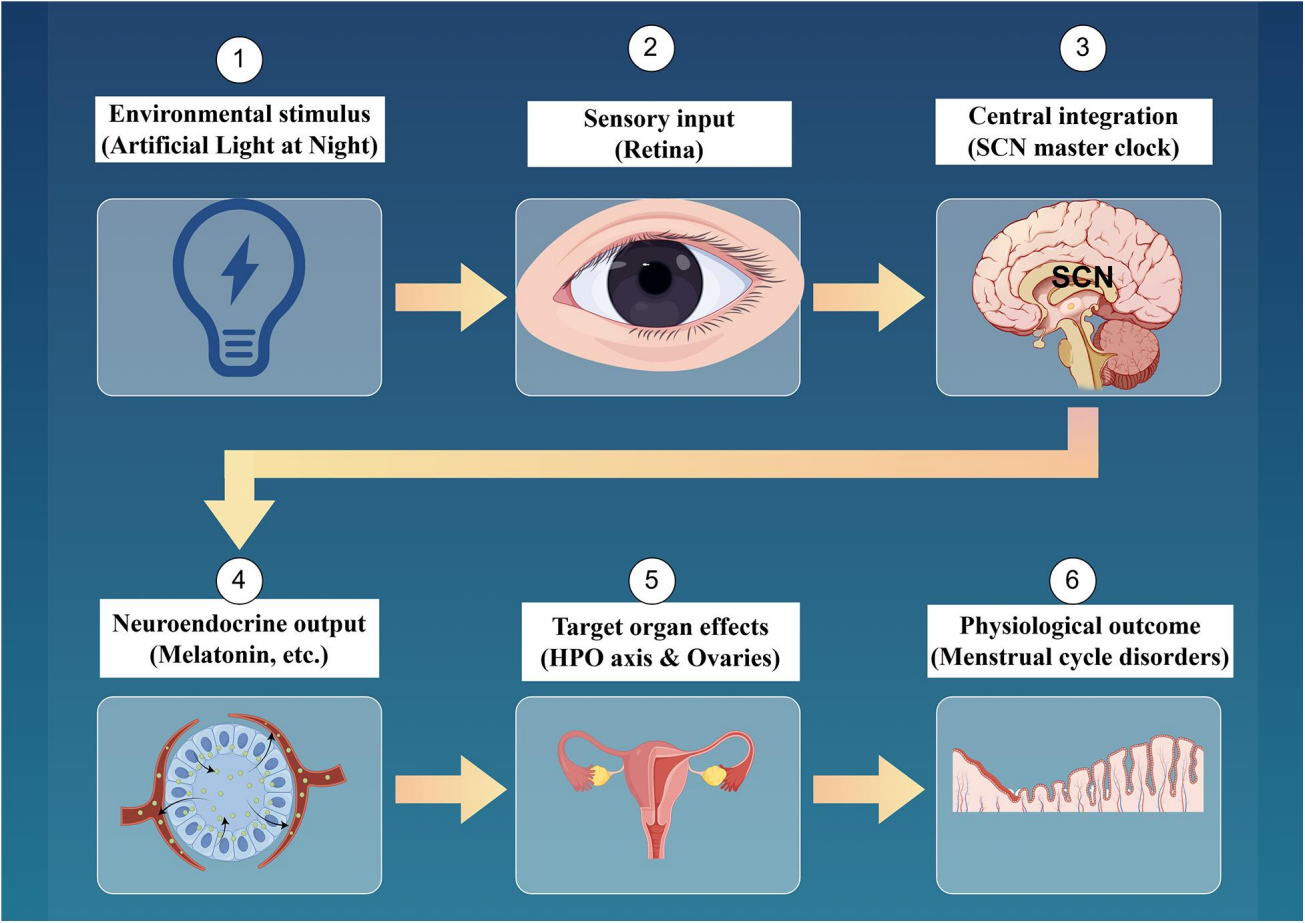
Overall mechanism diagram of menstrual cycle disorder caused by artificial light at night.

Women, as a significant demographic impacted by ALAN, warrant particular consideration concerning their physiological health. The menstrual cycle, a fundamental aspect of reproductive health, is primarily governed by the hypothalamic-pituitary-ovarian (HPO) axis, which relies on the precise synchronization of hormonal secretion and circadian rhythms (6, 7). The circadian rhythm is chiefly regulated by hormones such as melatonin, secreted by the pineal gland. Melatonin production is heavily influenced by light exposure, and ALAN can substantially suppress its secretion, thereby affecting subsequent hormonal regulation networks (1, 5). Moreover, artificial light sources, particularly those emitting blue light, are increasingly recognized as endocrine disruptors that can interfere with estrogen secretion, thereby elevating the risk of endocrine-related disorders in women (3). Epidemiological and experimental studies have indicated that artificial nighttime lighting may be associated with an increased incidence of breast cancer and menstrual disorders in women (2, 8) (Figure 1).

In this context, examining the effects of nighttime artificial lighting on women's menstrual cycles and developing appropriate care strategies is of considerable theoretical and practical importance. As light pollution increases, women are subjected to artificial light at elevated frequencies and intensities, thereby exacerbating associated health risks (9). Moreover, menstrual cycle irregularities not only impact women's reproductive health but may also serve as early indicators of various chronic diseases. Consequently, a systematic analysis of the mechanisms through which ALAN affects women's menstrual cycles, coupled with the proposal of evidence-based nursing interventions, can contribute to enhancing women's overall health.

This article aims to provide a critical review that synthesizes current evidence on the health risks of ALAN exposure, the physiological basis and regulatory mechanisms of the female menstrual cycles, and the role of light exposure in influencing reproductive health through circadian disruption. The paper first establishes a framework for evidence appraisal, followed by discussions of epidemiological findings and underlying biological mechanisms. It then focuses on practically oriented nursing intervention strategies. By integrating these perspectives, the review seeks to inform future research and clinical practice in this field.

Artificial Light at Night (ALAN), especially blue-rich light from screens and urban pollution, affects the intrinsically photosensitive retail ganglion cells (ipRGCs), which send signals to the brain's suprachiasmatic nucleus (SCN), disrupting the circadian clock. This disruption lowers melatonin and alters GnRH secretion, affecting the hypothalamic-pituitary-ovarian (HPO) axis. As a result, gonadotropin and sex hormone levels change, impacting ovarian and uterine functions, leading to menstrual disorders like irregular cycles and ovulatory issues. The figure illustrating this was created by the usage of Figdraw 2.0.

## Epidemiological status of ALAN and characteristics of female exposure

### Primary sources and trends in the proliferation of artificial lighting

With the rapid pace of urbanization and technological progress globally, artificial lighting has become a hallmark of modern nocturnal environment. Its primary sources include urban lighting (e.g., streetlights, billboards, and public illumination), residential indoor lighting, and various electronic screens (including televisions, computers, smartphones, etc.). The extensive use of urban lighting has profoundly transformed natural nighttime darkness, creating “light pollution.” Data indicate that over 80% of the global population resides in areas experiencing substantial light pollution, with these regions expanding at an annual rate of 2.2% (10). Concurrently, the prevalence of household lighting and electronic screens leads to continuous indoor exposure to ALAN. The widespread adoption of LED technology and display devices has markedly increased the duration and intensity of nighttime exposure (11, 12).

Exposure levels vary across populations. Epidemiological studies identify urban residents, night shift workers, and adolescents as susceptible groups. For example, urban women have a significantly higher likelihood of exposure to outdoor ALAN compared to their rural counterparts, directly linked to higher urban lighting density (13). Meanwhile, adolescents and young adults experience prolonged screen time at night due to frequent use of electronic devices, further elevating their exposure levels (14, 15).

In recent years, satellite remote sensing has provided a powerful tool for monitoring the global distribution and dynamics of ALAN. Research confirms that nocturnal light intensity in urban areas is significantly higher than in suburban and rural regions and continues to rise with urban expansion (16). Notably, the proliferation of new lighting technologies, such as LEDs, has altered the spectral composition of nighttime illumination, increasing the proportion of short-wavelength blue light, which may pose new risks to human health and ecosystems (11, 12).

In summary, exposure to ALAN originates mainly from urban/indoor lighting and electronic screens, with global exposure levels persistently increasing, particularly in urban and developed regions. Variations in exposure profiles across populations, influenced by environment and behavioral patterns. This information provides crucial data support for future research on health impacts and the development of intervention strategies.

### ALAN-induced circadian disruption in female subpopulations

Light exposure characteristics vary significantly among specific female subpopulations, influenced primarily by age, occupation, and lifestyle. Shift workers, especially nurses, experience prolonged exposure to artificial light at night due to occupational demands, which predisposes them to circadian disruption and subsequent adverse effects on endocrine and reproductive health. Research indicates that nocturnal light exposure during shift work significantly elevates the risk of endometrial cancer in female workers (17). Furthermore, this group frequently reports sleep disorders, menstrual irregularities, and long-term exposure may activate tumor-related signaling pathways.

Student population, particularly adolescents and college students, extendtheir exposure to short-wavelength blue light through increased nighttime screen time from electronic devices. Animal studies demonstrate that such exposure can disrupt normal sex hormone levels, induce ovarian inflammatory responses, and is associated with polycystic ovary syndrome-like alterations and follicular apoptosis, suggesting that the detrimental effects on the reproductive system intensify with prolonged exposure duration (18, 19).

Pregnant women represent another high-risk group. Nocturnal light exposure during pregnancy affects not only the mother but also fetal development via placental transmission. Animals studies confirm that chronic exposure can lead to maternal hormonal imbalances, abnormal uterine structure, and reduced antioxidant enzyme activity in female offspring, indicating potential long-term negative consequences for the development of subsequent generations’ reproductive system (20).

Age is also a critical factor. Middle-aged and elderly women exhibit heightened sensitivity to circadian disruption. Chronic exposure to nocturnal light in this group may contribute to immune abnormalities, reduced lifespan, and metabolic disorders, with women generally being more vulnerable to these adverse effects than men (21, 22).

**Figure 2:**
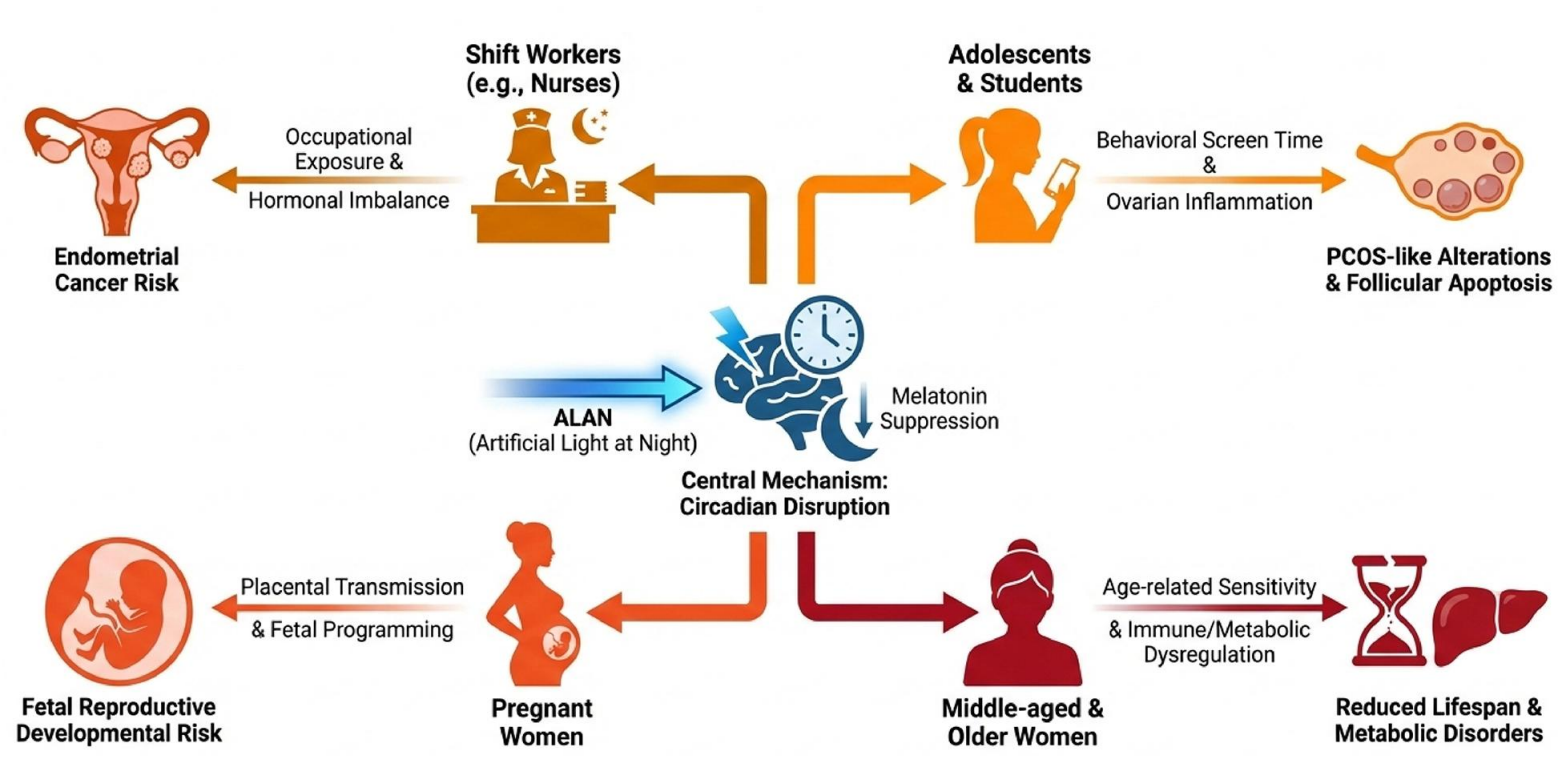
Divergent Reproductive health risks from ALAN-induced circadian disruption across female subpopulations.

In summary, occupational patterns (e.g., shift work) and behavioral habits (e.g., nighttime electronic device use) collectively determine women's light exposure profiles. Developing personalized light management and health intervention strategies tailored to specific subgroups—such as shift workers, pregnant women, adolescents, and the elderly—is crucial for mitigating associated reproductive health risks (Figure 2).

ALAN-induced circadian disruption acts as a central mechanism that differentially impacts female subpopulations based on occupation (shift work), behavior (screen use), and life stage (pregnancy, aging). This leads to distinct reproductive and metabolic health risks, including cancer, PCOS-like traits, fetal developmental issues, and reduced lifespan, necessitating targeted interventions. ALAN, artificial light at night; PCOS, polycystic ovary syndrome.

## The impact of ALAN on women’s menstrual cycles: epidemiological evidence

### Evidence assessment framework and limitation analysis

To systematically evaluate the strength of evidence regarding the association between artificial light at night (ALAN) exposure and women's menstrual health, this review categorizes existing evidence into the following four tiers based on study design, capacity for causal inference, and potential biases (23, 24):

Tier I (Strong Causal Inference): Derives from well-designed, large-scale prospective cohort studies (e.g., the Nurses’ Health Study) that adequately control for confounders and may demonstrate a dose-response relationship.

Tier II (Suggestive Associative Evidence): Derives from cross-sectional or case-control studies. While demonstrating statistical associations, the direction of causality remains uncertain, and findings are susceptible to confounding and recall bias.

Tier III Evidence (Ecological or Indirect Evidence): Includes correlations based on population-level data (e.g., regional light pollution data vs. menstrual disorder rates) or supportive evidence from animal models and mechanistic studies.

Tier IV (Expert Opinion/Preliminary Reports): Comprises case reports, small-sample preliminary studies, or theoretical inferences.

### Epidemiological features of menstrual cycle disorders

Menstrual cycle disturbances are prevalent among reproductive-aged women, encompassing irregularities in cycle length, bleeding volume, and dysmenorrhea. Specific environmental or physiological stressors can significantly increase their incidence, as evidenced by post-COVID-19 vaccination abnormalities such as heavy bleeding (24.24%), shortened cycles (16.2%), irregular cycles (6.6%), and oligomenorrhea (22.7%) (25). Key contributing factors also include: exposure to endocrine-disrupting chemicals (EDCs) (linked to irregular menstrual cycles, early menopause, and premature ovarian failure) (26–29); obesity(elevating risk via HPO axis disruption) (30); and functional hypothalamic amenorrhea (FHA) in athletes/adolescents due to excessive exercise or low weight (with 16%–47% having eating disorders and 32% menstrual dysfunction (31, 32). Furthermore, menstrual cycle irregularities are frequently accompanied by a spectrum of psychological and mental health issues, including anxiety, depression, insomnia, self-harm, and suicidal ideation in adolescents (33). Premenstrual syndrome (PMS) and premenstrual dysphoric disorder (PMDD) also significantly impairs women's social functioning and quality of life (34–37). Dysmenorrhea, another prevalent menstrual-related issue, is widespread and significantly impactful, with ∼30% of women reporting new/worsened symptoms during COVID-19 (38). Menstrual-related migraines (including both pure and associated types) are similarly common and tied to hormonal fluctuations (39–42). In conclusion, menstrual cycle disorders represent a prevalent issue within female reproductive health, characterized by a complex interplay of physiological, psychological, and environmental factors that significantly impact women's overall health and quality of life.

### Study on the correlation between artificial light exposure and menstrual cycle abnormalities

Recent research has increasingly focused on the influence of artificial light exposure on menstrual cycle irregularities. Table 1 summarized these critical findings on population level.

**Table 1:** Epidemiological evidence on the association between artificial light at night and menstrual disturbances: study designs, findings, and evidence strength assessment.

Study population/exposure type	Study design	Key findings	Evidence strength and limitations
Shift Workers (e.g., nurses, female factory workers)	Prospective cohort studies, cross-sectional studies	Increased risk of irregular menstruation, dysmenorrhea, and premenstrual syndrome; long-term exposure is associated with elevated risk of reproductive system diseases such as endometrial cancer (17).	Moderate to strong evidence (I-II). Large cohorts (e.g., Nurses’ Health Study) provide good temporal evidence. However, exposure is often intertwined with strong confounders like stress, sleep deprivation, and irregular eating patterns, making it difficult to isolate the independent effect of ALAN.
Adolescents and young students	Primarily cross-sectional studies	Positive correlation between nighttime electronic device use duration and risks of irregular menstruation and dysmenorrhea; may affect the pace of pubertal development (18).	Limited evidence (II-III). Most studies are cross-sectional, precluding causal inference. Screen time is highly collinear with academic stress, social media use, and sleep deprivation, making confounding control challenging.
General women of reproductive age	Ecological studies, cross-sectional studies	Higher residential outdoor ALAN levels are associated with shorter menstrual cycles and decreased regularity; weakens natural synchrony with the lunar cycle (43).	Suggestive evidence (III). Ecological studies reveal spatial associations at the population level but cannot infer individual causality. Exposure measurement based on satellite data differs from individual actual exposure.
Pregnant women	Primarily animal studies, very limited human data	Maternal ALAN exposure may affect the development of the offspring's reproductive system, potentially linked to abnormal hormone levels and altered uterine function in offspring (20).	Mechanistic exploration stage (III-IV). Human epidemiological evidence is almost absent. Animal models provide important biological plausibility, indicating a direction warranting further research.

ALAN, artificial light at night.

### Shift work: occupational nighttime light exposure

Shift work, particularly occupations requiring prolonged nighttime work, provides a “natural experiment” for studying the health effects of ALAN. Multiple studies consistently show that the incidence of menstrual disturbances is significantly higher among female shift workers compared to daytime workers. For instance, research on nurses indicates that those with higher frequency and longer duration of night shifts report greater proportions of irregular menstrual cycles, abnormal bleeding, and severe dysmenorrhea (17). The biological plausibility of this association lies in the fact that shift work forcibly displaces activity, eating, and light exposure into the biological clock's “rest phase,” leading to desynchronization between the rhythmic secretion of HPO axis hormones (e.g., LH pulses) and behavioral cycles. Furthermore, some long-term follow-up studies suggest that decades of night shift work may be associated with an increased risk of hormone-related cancers such as endometrial and breast cancer, although these findings require further confirmation, and carcinogenesis is the result of long-term multifactorial influences (8, 17).

### Screen time and residential light exposure: exposure in daily life

For non-shift working populations, ALAN exposure primarily originates from indoor lighting and electronic screens. Cross-sectional surveys suggest that women who self-report prolonged use of smartphones, computers, or tablets before bedtime appear to have a higher risk of experiencing irregular menstruation and dysmenorrhea. However, such studies are significantly affected by recall bias, and screen time is highly correlated with staying up late, psychological stress, and poor lifestyle habits, complicating the independent assessment of the effect of screen-based blue light (2). In recent years, studies utilizing high-resolution satellite data to assess community-level nighttime light levels have found that women living in areas with higher outdoor ALAN brightness may have shorter average menstrual cycle lengths and poorer cycle regularity (43). Such ecological studies provide a macro-level perspective on the potential impact of environmental light but cannot confirm causality at the individual level.

### Confounding and bias control in association studies

Interpreting the association between ALAN and menstrual disturbances requires caution. Key confounding factors include: (1) Sociodemographic and lifestyle factors: age, BMI, smoking, alcohol consumption, physical exercise, and dietary patterns (44–46). For example, obesity itself is a strong risk factor for menstrual disturbances and may correlate with certain ALAN exposure patterns (30); (2) Psychosocial factors: stress, anxiety, and depression, which are both triggers for menstrual disturbances and potentially related to nighttime wakefulness and electronic device use (47); (3) Direct reproductive factors: The use of hormonal contraceptives, which artificially regulate the menstrual cycle, is a critical variable that must be controlled (48). (4) Sleep factors: Sleep duration and quality are central mediators linking light exposure to health outcomes but also constitute strong confounding factors themselves.

Ideal studies should employ objective methods to measure individual light exposure (e.g., wearable photometers), prospectively collect detailed menstrual cycle data (e.g., via period-tracking apps) (49, 50), and utilize advanced statistical models (e.g., time-dependent models, mediation analysis) to control for confounders and explore mechanistic pathways (51). Currently, such high-quality studies remain insufficient.

## The biological underpinnings of light exposure: intensity, Spectrum, and timing

**Figure 3:**
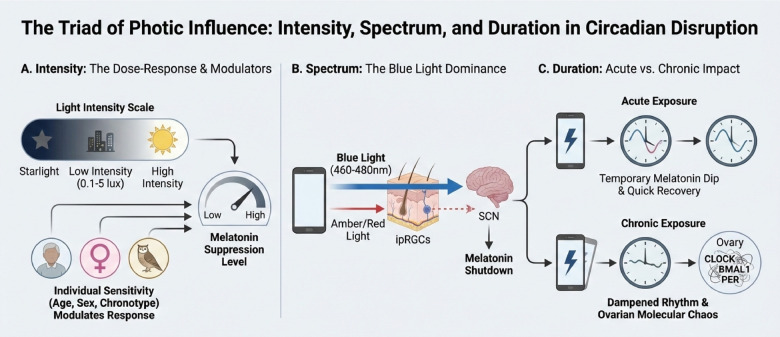
Distinct physical dimensions of light exposure drive circadian disruption. **(A)** Intensity and individual facors modulate melatonin suppression; **(B)** blue light is the dominant spectral regulator of the SCN; **(C)** acute exposure causes temporary disruption, while chronic exposure leads to persistent dysregulation and molecular chaos in peripheral tissues like the ovary. SCN, suprachiasmatic nucleus.

Light is not merely illumination; it is the primary environmental cue that synchronizes our internal biological clocks. The physiological impact of artificial light at night hinges critically on its physical properties—intensity, spectral composition, and duration of exposure. These parameters act through distinct yet interconnected pathways to disrupt the delicate orchestration of the circadian system, with downstream consequences for reproductive endocrinology (Figure 3).

### Light intensity: a dose-dependent effect on melatonin inhibition and circadian rhythms

Light intensity, as a crucial environmental factor, exerts a profound influence on the physiology and behavior of organisms, particularly in the regulation of melatonin secretion and circadian rhythms (1, 52). Seminal work has demonstrated that even exceedingly dim light exposure at night—as low as 0.1–5 lux, comparable to the faint glow of a city sky or a nightlight—can exert measurable physiological effects. At these low levels, nocturnal melatonin secretion is partially suppressed, and subtle shifts in circadian phase may occur (53). This finding establishes a fundamental principle: there exists no truly “safe” or biologically insert intensity of nighttime light for the circadian system. As intensity escalates, so does the potency of its effects. Exposure to typical indoor room light (>500 lux) can induce near-complete melatonin suppression (54), while brighter light of ∼10,000 lux (approximating daylight) not only suppresses melatonin profoundly but also elicits robust phase shifts of the circadian clock (55). The mechanics of these phase delays are linked to light's direct resetting action on the suprachiasmatic nucleus during the late biological evening (56).

However, this dose-response relationship is not uniform. Inter-individual sensitivity to light intensity is modulated by intrinsic factors: older adults often exhibit heightened melatonin suppression (57); emerging evidence suggests that women may experience stronger melatonin suppression and phase-shifting responses than man under 400-2000 lux lighting (58, 59); and one's inherent chronotype further refines this personal sensitivity profile (60). Consequently, defining an objectively “bright” or “disruptive” light is context-dependent, requiring integration of both the ambient photic dose and the individual's physiological state.

It is crucial to note that direct epidemiological evidence linking quantified environmental *light intensity* to menstrual disorders remains sparse. The primary human evidence stems from studies on night shift workers, whose environments invariably involve high-intensity lighting. Thus, these observed associations cannot be disentangled from the potent confounders of circadian misalignment, sleep deprivation, and psychosocial stress inherent to shift work. Well-designed prospective population studies directly investigating the relationship between objectively measured light intensity and menstrual cycle parameters constitute an important gap for future research.

### Spectral composition: the pivotal role of short-wavelength blue light

Various artificial light sources exert distinct regulatory effects on circadian clocks, particularly in nocturnal lighting environments. Blue light, characterized by short wavelengths (approximately 460–480 nm), has been shown to possess the most potent capacity to synchronize and disrupt circadian rhythms in both humans and animals. This effect is mediated through the activation of intrinsically photosensitive retinal ganglion cells (ipRGCs) in the retina, which enhances the suprachiasmatic nucleus's (SCN) perception of external light cues, thereby influencing melatonin secretion and the expression of circadian clock genes. Empirical evidence suggests that exposure to blue light or white light with a high correlated color temperature (CCT) during nighttime significantly suppresses melatonin production, resulting in delayed circadian rhythms, diminished sleep quality, and circadian rhythm disorders (61, 62). In stark contrast, longer wavelengths such as amber or red light engage this pathway far less effectively, resulting in markedly weaker circadian and melatonin responses (63). Furthermore, the findings indicate that both light intensity and spectral composition collectively modulate the impact on circadian rhythms, with high-intensity and highCCT illumination at night more effectively suppressing nocturnal activity and melatonin secretion (64, 65). It is important to note that the white light frequently employed in contemporary lighting technologies, such as LEDs, contains a substantial proportion of blue light, thereby elevating the risk of biological clock disruption, particularly when utilized during nighttime (52, 66). This exposure shift means that even at moderate intensities, contemporary nighttime lighting can be disproportionately disruptive due to its spectral signature, making the composition of light as critical a parameter as its brightness. Although further studies have proposed that blue light exposure and hormonal imbalance may be associated with female reproductive health (67), the relevant direct evidence remains extremely limited. Future research should prioritize large-scale prospective studies to elucidate the causal relationship and underlying mechanisms between blue light exposure and menstrual cycle disruptions. This will provide evidence-based support for the development of scientifically grounded prevention and care strategies.

### Exposure duration: acute disruption versus chronic desynchrony

The temporal pattern of light exposure dictates whether its effects are transient or lead to enduring circadian pathology. Acute, short-term exposure—an occasional late night spent in front of a screen, for instance—can cause a temporary dip in melatonin and fragment sleep (68, 69). The system typically recovers once natural darkness is restored. Chronic exposure, however, poses a far greater threat. Persistent nighttime illumination, as experienced in long-term shift work, does not merely perturb the circadian system; it can fundamentally degrade its function. Over time, the robust rhythmic output of the SCN dampens. The amplitude of melatonin secretion diminishes, its peak may drift, and the precise timing of hormonal signals becomes erratic. This state of internal desynchrony is mirrored at the molecular level within downstream hormone axis [eg., hypothalamic-pituitary-ovarian (HPO) axis and hypothalamic-pituitary-adrenal (HPA) axis] and peripheral tissues, including the ovaries (70–72). Core clock genes such as *Bmal1* and *Per* lose their coherent 24-hour oscillation patterns. This molecular chaos within reproductive tissues is not a mere correlate but a likely direct contributor to impaired folliculogenesis, ovulation, and uterine function. More seriously, chronic circadian disruption may further contribute to emotional disorders and cognitive decline (73), as well as increase the risk of tumor and metabolic syndrome (74). Thus, while intensity and spectrum determine the potency of the light signal, duration ultimately determines the severity and persistence of the circadian insult.

## Molecular and neuroendocrine mechanisms of artificial light exposure on menstrual cycles

The impact of ALAN on the menstrual cycle, mediated primarily through the disruption of neuroendocrine regulation, involves core mechanistic pathways as outlined in Table 2 and detailed in the text below.

**Table 2:** Summary of proposed mechanistic pathways linking ALAN to menstrual cycle disruption.

Mechanism tier	Key components & processes	Primary consequences	Link to menstrual outcomes
Signal input & central processing	Retina: ipRGCs are preferentially activated by short-wavelength (blue) light. SCN: Altered light signaling disrupts the master circadian pacemaker.	Suppression of pineal melatonin secretion; generation of erroneous or dampened circadian timing signals.	Creates the foundational neuroendocrine disruption that desynchronizes downstream reproductive axes.
Neuroendocrine axis dysregulation	HPO Axis: Reduced melatonin and aberrant SCN output impair pulsatile GnRH release, leading to abnormal LH/FSH secretion. HPA Axis (potential): ALAN may act as a chronic stressor, elevating cortisol, which can further suppress reproductive function.	Disrupted cyclicity of gonadotropins and ovarian sex hormones (estrogen, progesterone).	Directly leads to anovulation, luteal phase defects, and irregular follicular development, manifesting as irregular cycles.
Peripheral clock & cellular dysfunction	Ovarian/Uterine Clocks: Desynchrony of core clock genes (e.g., *Bmal2*, *Per1/2*) impairs local rhythmic functions. Cellular Stress: Loss of melatonin's antioxidant protection and activation of stress pathways (e.g., PKC-*α*/Akt).	Impaired folliculogenesis, steroidogenesis, uterine receptivity, and increased oxidative stress/inflammation in reproductive tissues.	Contributes to subfertility and creates a tissue microenvironment conducive to long-term pathology.
Long-term pathophysiological potential	Sustained Disruption: Chronic circadian misalignment and hormonal imbalance. Experimental Evidence: Activation of proliferative/apoptotic pathways in animal models.	Increased risk of endometrial hyperplasia, endometriosis-like phenotypes, and (in extreme experimental models) endometrioid adenocarcinoma.	Provides biological plausibility for epidemiological associations between chronic night shift work and severe reproductive disorders.

ALAN, artificial light at night; ipRGCs, intrinsically photosensitive retinal ganglion cells; SCN, suprachiasmatic nucleus; GnRH, gonadotropin-releasing hormone; HPO axis, hypothalamic-pituitary-ovarian axis; LH, luteinizing hormone; FSH, follicle-stimulating hormone; HPA axis, hypothalamic-pituitary-adrenal axis.

### Core pathway: melatonin suppression and HPO axis dysregulation

Melatonin, a critical hormone produced by the pineal gland during nighttime, is profoundly influenced by environmental light cycles, with levels markedly increasing during the night and decreasing throughout the day. Nighttime light exposure, especially blue light, strongly suppresses pineal melatonin secretion via the ipRGCs-SCN pathway. Melatonin is not merely the “sleep hormone”, it is also a crucial regulator of the reproductive system. By acting on MT1 and MT2 receptors located on hypothalamic GnRH neurons, the pituitary gland, and the ovaries, it participates in regulating pulsatile GnRH secretion, the release of gonadotropins [luteinizing hormone (LH) and follicle-stimulating hormone (FSH)], and ovarian steroidogenesis (75, 76).

**Figure 4:**
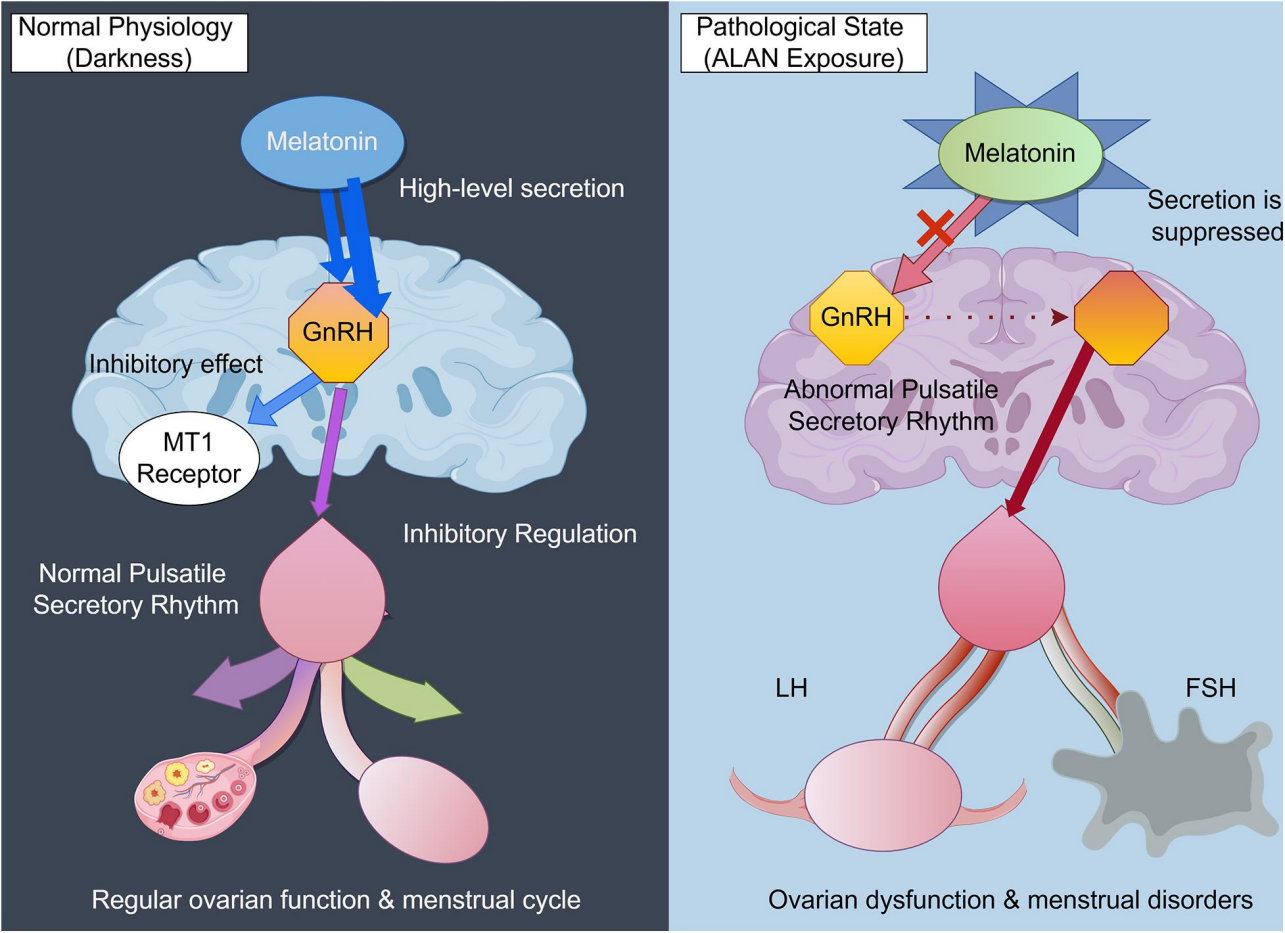
The molecular mechanism by which melatonin regulates the HPO axis and Its disruption by ALAN.

Melatonin exerts protective effects on ovarian cells by acting as an antioxidant, thereby preserving their optimal physiological condition, which is essential for reproductive health (75). Animal experiments have confirmed that artificial suppression or disruption of melatonin rhythms leads to estrous cycle irregularities, ovulation disorders, and reduced fertility (77) (Figure 4). The menstrual disturbances observed in female shift workers may be related to this light-induced disruption of melatonin rhythms and its subsequent interference with the HPO axis.

In summary, melatonin plays a pivotal role in regulating female reproductive hormone secretion and the menstrual cycle rhythm through its complex interactions with the hypothalamic-pituitary-ovarian (HPO) axisEnvironmental light exposure, patterns of melatonin secretion, and the dynamics of receptor expression collectively contribute to the maintenance of endocrine balance and physiological functions within the female reproductive system (78, 79). A more profound understanding of melatonin's regulatory mechanisms could offer new theoretical foundations and intervention strategies for safeguarding reproductive health and preventing associated disorders.

In low-light conditions, elevated melatonin levels activate the MT1 receptor, inhibiting Gonadotropin-Releasing Hormone (GnRH) release from the hypothalamus. This inhibition maintains the normal rhythmic secretion of GnRH, luteinizing hormone (LH), and follicle-stimulating hormone (FSH), essential for regular ovarian function and the menstrual cycle. However, artificial light at night (ALAN) suppresses melatonin, removing this inhibition and disrupting the secretion rhythm of GnRH, leading to irregular LH and FSH release. This disruption results in ovarian dysfunction and menstrual disorders. The accompanying figure was created by the author using Figdraw.

### Disruption of clock genes: desynchrony at the molecular level

The SCN, as the master circadian clock, derives its rhythm from a transcription-translation feedback loops (TTFLs) driven by a series of clock genes (e.g., *CLOCK*, *BMAL1*, *Per*, *Cry*) (80). At the molecular level, the *CLOCK* and *BMAL1* genes sustain a 24-hour circadian rhythm through TTFLs. ALAN disrupts the expression rhythms of these genes within the SCN, leading to inaccurate or dampened timing signals from its output and, in some cases, a complete loss of circadian patterns (81, 82). More importantly, animal studies have corroborated that continuous or low-intensity nighttime light significantly suppresses the rhythmic expression of clock genes, including *Per1*, *Per2*, and *Nr1d1*, within the SCN, thereby disrupting hormone secretion and metabolic status through effects on the downstream endocrine axis (81, 83). Furthermore, animal researches indicates these clock genes expression is also impacted by light intensity, spectrum, and duration (84–86).

**Figure 5:**
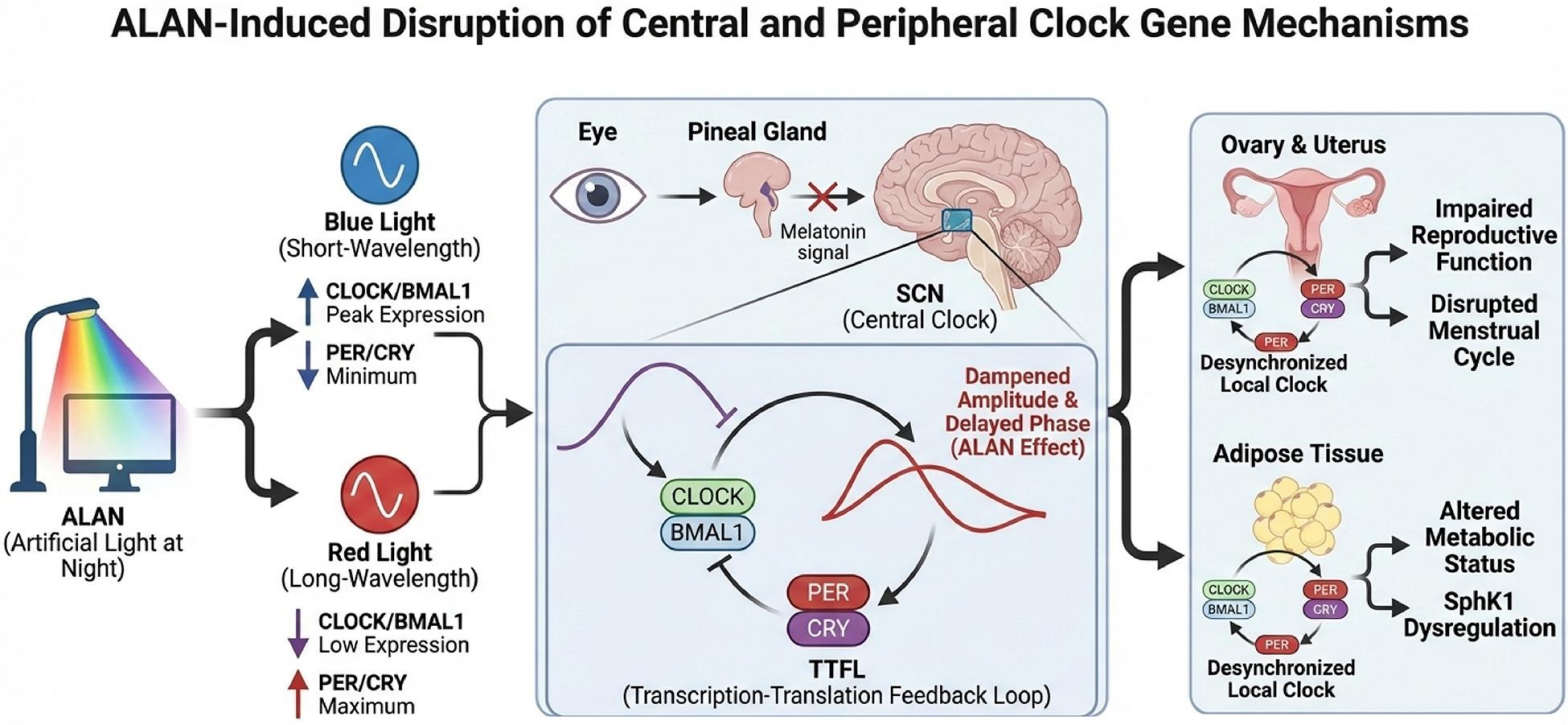
ALAN-Mediated disruption of central and peripheral clock gene mechanisms.

Peripheral organs, such as ovarian, were also evidenced to exhibit rhythmic expression of clock genes, which regulates key events such as follicular development, ovulation, and luteal function Long-term nighttime light exposure may lead to desynchrony between the SCN and ovarian clocks, as well as among different cell types within the ovary, thereby directly impairing reproductive function. Animal studies directly demonstrate that constant light exposure causes abnormal rhythmic expression of genes like *Bmal1* in the ovaries and uterus, accompanied by uterine inflammation, reduced receptivity, and worsened reproductive outcomes (82) (Figure 5).

Exposure to artificial light at night (ALAN), particularly blue wavelengths, alters core clock gene stoichiometry (*CLOCK/BMAL1* vs. *PER/CRY*) and disrupts the central SCN pacemaker. This central desynchrony suppresses melatonin and misaligns peripheral clocks in ovarian and adipose tissues, ultimately driving reproductive impairment and metabolic dysregulation.

### From mechanism to disease: extending experimental evidence

In-depth animal mechanistic studies provide clues for understanding the potential long-term risks posed by ALAN. For instance, the study by Das et al. showed that long-term exposure to light cycles simulating shift work could induce endometriosis-like lesions and ultimately lead to endometrioid adenocarcinoma in hamsters, involving the persistent activation of the PKC-*α*/Akt signaling pathway and inhibition of apoptosis (17). Another study by Das et al. found that constant light exposure, by disrupting central and ovarian clocks and reducing antioxidant enzyme activity, impaired uterine angiogenesis and embryo implantation capacity (82). Furthermore, neuroendocrine factors such as kisspeptin and neurokinin B (NKB) have been identified as regulators of GnRH in the pathogenesis of polycystic ovary syndrome (PCOS). The hyperactivity of the kisspeptin system is associated with the overactivation of the HPO axis, which further exacerbates menstrual irregularities and hyperandrogenemia (87).

Beyond the HPO axis, the HPA axis and the hypothalamic-pituitary-thyroid (HPT) axis are also intricately linked to the menstrual cycle. Chronic stress can activate the HPA axis, resulting in elevated cortisol secretion. Sustained high cortisol levels may compromise the function of the reproductive axis, leading to hormonal imbalances and menstrual disturbances. Similarly, thyroid dysfunction can affect the pituitary gland's secretion of FSH and LH, thereby impacting ovarian function (39, 88).

Moreover, modifications in neurotransmitters and gut microbiota significantly influence neuroendocrine regulation. In patients with PCOS, the increased production of gamma-aminobutyric acid (GABA) by gut bacteria is associated with elevated levels of LH and an increased LH/ FSH ratio, indicating that the gut-brain-gonadal axis may play a critical role in menstrual irregularities (89). On the other hand, gut-microbiota composition alteration is correlated with both central and peripheral clock genes expression in anorexia mice model (90). Additionally, in pregnant mice model, peripheral reproductive clock genes are reported to affect uterine physiology via Akt/FoxO1 pathway (91), and prostaglandin G/H synthase 2 (PTGS2) upregulating (92).

Although this experimental evidence cannot be directly equated with human disease processes, it strongly suggests that long-term, severe circadian disruption may increase the risk of pathological changes in the female reproductive system through specific molecular pathways. This provides biological plausibility for the weak signals observed in epidemiology regarding the link between night shift work and the risk of certain gynecological cancers.

Current understanding of the mechanisms by which ALAN affects the menstrual cycle relies heavily on animal models and *in vitro* studies. While this research has revealed highly conserved physiological pathways (e.g., the ipRGC-SCN-melatonin axis), significant limitations exist: (1) Species differences: Human reproductive endocrine regulation is more complex and more sensitive to psychosocial factors; (2) Exposure simulation differences: Animal experiments often use extreme (e.g., constant light) or single-wavelength light conditions, which differ from the complex, intermittent, and polychromatic exposure patterns in real human life; (3) Endpoint differences: Endpoints in animal studies are often estrous cycles, hormone levels, or ovarian morphology, which are not fully equivalent to subjectively reported menstrual symptoms or clinically diagnosed menstrual disorders in humans. Therefore, when extrapolating this mechanistic evidence to human menstrual health, it is crucial to recognize its suggestive rather than conclusive role. Future research needs to incorporate biomarker measurements (e.g., salivary melatonin, cortisol rhythms) within human prospective studies alongside menstrual outcomes to validate the relevance of these mechanistic pathways in human populations.

## Nursing interventions and public health implications

Based on current evidence, nursing practice can intervene at the individual, occupational, and policy levels to prevent and mitigate the potential impact of ALAN on women's menstrual health.

### Nursing assessment and personalized management

Nurses play a key role in clinical and community health services. First, a history of ALAN exposure should be incorporated into routine women's health assessments, including inquiries about: occupational nature (e.g., shift work), years of night work, electronic screen use habits 1–2 hours before bedtime, bedroom sleep environment brightness, and light source color. Second, women should be encouraged and guided to use menstrual cycle tracking tools (e.g., apps, diaries) to record cycle length, regularity, and menstrual symptoms over the long term, comparing these records with light exposure behaviors to raise self-health awareness. For women who already experience menstrual disturbances suspected to be related to light exposure, nursing interventions may include (93, 94):

(1) Health Education: Explain the basic principles of how light affects circadian rhythms and hormones, emphasizing the importance of protecting a dark environment at night. Healthcare providers should eschew stereotypes, actively engage with patients’ individual experiences, and deliver gender-affirming health services to mitigate psychological distress (95, 96).
(2) Behavioral Guidance: Develop personalized “light hygiene” plans, such as setting a “screen curfew,” using warm-toned reading lights before bed, and ensuring the bedroom is completely dark.
(3) Special Population Focus: For shift-work nurses, guide them to wear blue-light-blocking glasses on their way home after a night shift and use blackout curtains for daytime sleep to promote rhythm resetting. In the context of adolescents and college students, nursing assessments should extend beyond physiological symptoms to include evaluations of menstrual knowledge, hygiene management skills, and challenges associated with educational settings and cultural barriers (97, 98).

### Behavioral and lifestyle interventions

Evidence-based protective behaviors and environmental interventions that individuals can adopt include:

(1) Optimizing the Nighttime Light Environment: 1–2 hours before bedtime, switch main indoor lighting to low-brightness (<30 lux), low-color-temperature (<2700 K) warm light. Avoid using strong cool-white LED ceiling lights.
(2) Managing Screen Time: When using electronic devices at night, enable “eye protection” or “night shift” modes (which reduce blue light output) and minimize screen brightness as much as possible. Avoid viewing screens up close in complete darkness (99, 100).
(3) Maintaining Sleep Hygiene: Keep relatively fixed bedtimes and wake-up times, even on weekends. Create a dark, quiet, and cool sleep environment, using blackout curtains and eye masks if necessary.
(4) Focusing on Overall Health: Managing stress, maintaining a healthy weight, eating a balanced diet, and exercising regularly all help stabilize the endocrine system and enhance resilience to environmental stressors, including light exposure.

### Occupational health and policy advocacy

Systemic measures are needed for occupationally exposed groups:

(1) Workplace Health Promotion: Employers should provide education for shift workers on the health risks of light exposure and consider optimizing workplace lighting (e.g., reducing light intensity and blue light content in the latter half of a night shift). Offer health screenings and include menstrual health in occupational health monitoring.
(2) Policy Research and Advocacy: Public health departments should support further research on the health effects of ALAN, particularly long-term follow-up studies. Urban planning should consider “light pollution” control and develop community nighttime lighting standards. Integrate the concept of “healthy lighting” into architectural design and public health guidelines.

In conclusion, addressing the impact of nighttime artificial lighting on women's menstrual cycles necessitates a collaborative approach across multiple disciplines and the systematic implementation of policies. Effective mitigation of the adverse effects of nighttime illumination on reproductive health and the enhancement of overall women's well-being can only be achieved through coordinated efforts involving healthcare, gynecology, public health, and related sectors, alongside the development of well-designed lighting policies and comprehensive public awareness campaigns. We propose a series of comprehensive intervention strategies in this chapter, as summarized in Table 3.

**Table 3:** Tiered intervention strategies to mitigate the potential impact of ALAN on menstrual health.

Intervention level	Specific strategies	Objectives and rationale
Individual/household level	Pre-sleep Light Management: Use warm light (<2700 K), reduce brightness. Screen Use Management: Enable blue-light filtering functions, reduce pre-sleep usage duration. 3. Sleep Environment Optimization: Use blackout curtains to ensure a dark bedroom.	Reduce retinal light signal input, promote normal endogenous melatonin secretion, and stabilize the circadian clock.
Behavioral promotion level	Regular Schedule: Maintain fixed sleep-wake times. Health Education: Disseminate knowledge on ALAN health risks to improve self-care capacity. 3. Symptom Monitoring: Encourage the use of menstrual tracking apps for early detection of abnormalities.	Reinforce endogenous rhythm stability, enhance self-management capability, and enable early detection and intervention.
Occupational/policy level	Support for Shift Workers: Develop personalized light management plans (e.g., post-shift blue-light protection), provide health screenings. 2. Policy Advocacy: Promote “healthy lighting” guidelines in workplaces, integrate reproductive health into the occupational health protection system.	Systematically reduce the negative impact of occupational ALAN exposure and protect the reproductive health of high-risk women at a structural level.

ALAN, artificial light at night; MCTAs, menstrual cycle tracking applications.

## Conclusion

ALAN is an emerging environmental health determinant, and its potential impact on women's menstrual cycles and reproductive health is attracting increasing attention. Current evidence suggests epidemiological associations between ALAN exposure (particularly shift work and nighttime screen use) and menstrual cycle disturbances. The potential mechanisms involve the suppression of melatonin secretion via the ipRGCs-SCN pathway, leading to dysregulation of HPO axis function and disruption of circadian clock gene expression in the ovaries and uterus. Animal experiments further reveal molecular pathways through which long-term circadian disruption may lead to impaired reproductive function and even pathological changes.

However, the limitations of existing research must be clearly recognized. Most human evidence is observational and associative, heavily confounded by factors such as stress, sleep, and lifestyle; definitive causal relationships have not been established. Exposure measurement methods are inconsistent, and data on long-term health outcomes are lacking. Translation from animal mechanisms to complex human contexts requires caution.

Future research should prioritize well-designed prospective cohorts combining objective, individualized light monitoring with detailed assessments of reproductive health endpoints. Experimental studies are needed to explore the acute and chronic effects of different light parameters (intensity, spectrum, pattern) on human endocrine function and menstruation. Interdisciplinary collaboration (encompassing environmental science, endocrinology, sleep medicine, nursing, and public health) is essential for a comprehensive understanding of this complex issue.

For nursing practice, sufficient biological plausibility and preliminary epidemiological evidence already exist to support integrating “light health” education into women's health promotion and providing targeted assessment and intervention guidance for high-risk women (e.g., shift workers). Through multi-level efforts—including individual behavior change, occupational health protection, and public health policy advocacy—the potential burden of ALAN on women's reproductive health can be mitigated, promoting overall well-being.
